# Targeting the Leukemia Inhibitory Factor/Leukemia Inhibitory Factor Receptor Axis Reduces the Growth of Inflammatory Breast Cancer by Promoting Ferroptosis

**DOI:** 10.3390/cancers17050790

**Published:** 2025-02-25

**Authors:** Bianca Romo, Zenaida Fuentes, Lois Randolph, Megharani Mahajan, Emily J. Aller, Behnam Ebrahimi, Bindu Santhamma, Uday P. Pratap, Panneerdoss Subbarayalu, Harika Nagandla, Christoforos Thomas, Hareesh B. Nair, Ratna K. Vadlamudi, Suryavathi Viswanadhapalli

**Affiliations:** 1 Department of Obstetrics and Gynecology, University of Texas Health San Antonio, San Antonio, TX 78229, USA; romob1@uthscsa.edu (B.R.); zenaida.fuentes@vanderbilt.edu (Z.F.); randolphl@uthscsa.edu (L.R.); mahajanm@uthscsa.edu (M.M.); aller@livemail.uthscsa.edu (E.J.A.); ebrahimib1@livemail.uthscsa.edu (B.E.); pratap@uthscsa.edu (U.P.P.); 2 Evestra, Inc., San Antonio, TX 78245, USA; bsanthamma@evestra.com; 3 Mays Cancer Canter, University of Texas Health San Antonio, San Antonio, TX 78229, USA; subbarayalu@uthscsa.edu; 4 Greehey Children Cancer Research Institute, University of Texas Health San Antonio, San Antonio, TX 78229, USA; 5 Houston Methodist Neal Cancer Center, Houston Methodist Research Institute, Houston, TX 77030, USA; hnagandla@houstonmethodist.org (H.N.); thomaschristoforos@gmail.com (C.T.); 6 Audie L. Murphy Division, South Texas Veterans Health Care System, San Antonio, TX 78229, USA

**Keywords:** LIF, LIFR, IBC, EC359

## Abstract

**Background:** Inflammatory breast cancer (IBC) is a rare subtype of breast cancer accounting for 7% of breast cancer-related fatalities. There is an urgent need to develop new targeted treatments for IBC. The progression of IBC has been associated with alterations in growth factor and cytokine signaling; however, the function of the LIF (leukemia inhibitory factor)/LIFR (leukemia inhibitory factor receptor) cytokine pathway in the progression of IBC remains unknown. This study evaluated the role of LIFR signaling and tested the efficacy of the LIFR inhibitor EC359 in treating IBC. **Methods:** The utility of using LIFR inhibition as a treatment strategy in IBC was tested using cell survival, apoptosis, colony formation, invasion, and pre-clinical KPL4 xenografts. Western blotting, siRNA, RT-qPCR, and lipid peroxidation assays were used to establish the mechanism of EC359 therapy. **Results:** The reduction in LIFR levels using siRNA markedly decreased growth in colony formation assays and reduced the invasion of IBC cells. Pharmacological inhibition of LIFR with EC359 effectively reduced cell survival and the clonogenic capacity of IBC cells. RT-qPCR assays revealed that EC359 markedly decreased the expression of the LIFR target genes. Western blot analyses confirmed that EC359 treatment suppressed downstream LIF/LIFR signaling pathways and promoted apoptosis. Treatment of cells with the ferroptosis inhibitor Fer-1 negated the capacity of EC359 to induce apoptosis. Mechanistic investigations demonstrated that EC359 predominantly triggered ferroptosis by inhibiting the glutathione antioxidant defense system through the downregulation of Glutathione peroxidase 4 (GPX4) levels. EC359 (5 mg/kg/day) was effective in reducing the growth of the IBC KPL4 xenograft tumors. **Conclusion:** These findings demonstrates that LIFR inhibition promote ferroptosis-mediated cell death in IBC and that EC359 represent novel therapeutic for IBC treatment.

## Simple Summary

Inflammatory breast cancer (IBC) is a rare subtype of breast cancer that lacks effective targeted therapies. Alterations in growth factor and cytokine signaling have been associated with the progression of IBC. The LIF (leukemia inhibitory factor)/LIFR (leukemia inhibitory factor receptor) cytokine signaling pathway has been linked to the progression of a variety of cancers; however, its importance in IBC has yet to be determined and was investigated in this study. The reduction in LIFR levels using siRNA significantly diminished the proliferation and invasion of IBC cells. The pharmacological inhibition of LIFR using EC359 significantly decreased the cell viability, clonogenicity, and invasive potential of IBC cells, inhibited LIF/LIFR downstream signaling, and enhanced apoptosis. Mechanistic studies revealed that EC359 primarily induced ferroptosis and effectively inhibited the growth of IBC xenograft tumors. Our research indicates that LIFR inhibition facilitates ferroptosis-mediated cell death in IBC, positioning LIFR inhibitors as a possible targeted therapy for IBC.

## 1. Introduction

Breast cancer (BC) is the most frequently diagnosed malignancy in women in the United States [1]. BC is classified into three main subtypes based on hormone receptor expression: estrogen receptor (ER), progesterone receptor (PR), and human epidermal growth factor receptor 2 (HER2). Inflammatory breast cancer (IBC) is an exceedingly rare type of breast cancer, with 2–4% of women diagnosed within this BC patient population [2]. IBC is characterized by aggressive behavior and rapid progression from the onset of the disease [3]. IBC can manifest as any of the three BC subtypes; however, IBC is more frequently observed as HER2-positive or triple-negative due to its aggressive nature [4]. Radiation, chemotherapy, and surgery are currently used as the standard care for treating IBC. Nevertheless, the survival rate remains low with a 5-year survival rate of approximately 40% [5]. At present, there are no FDA-approved targeted therapies for the treatment of IBC and new targeted therapies are urgently needed.

Numerous recent investigations have attempted to identify genes and pathways that are unique to IBC [6,7,8]. A target discovered in this molecular profiling was IL-6, a pro-inflammatory cytokine responsible for activating the JAK/STAT3 pathway [8]. The research conducted by Stevens et al. in 2023 also emphasized the significance of JAK/STAT3 signaling in the proliferation of IBC and its response to treatment [9]. The leukemia inhibitory factor (LIF) is the most pleiotropic member of the IL-6 family. The LIF activates its downstream signaling via binding to the heterodimeric complex of leukemia inhibitory factor receptor (LIFR) and glycoprotein 130 (gp130), which lacks intrinsic tyrosine kinase activity and depends on its persistent interaction with JAK/STAT members for signaling activation [10]. LIFR has previously been linked to the progression of BC [11,12], but its role in IBC progression has not yet been explored. We hypothesize that the LIF/LIFR axis could be involved in the progression of IBC, and the genetic or pharmacological inhibition of LIFR could be a therapeutic strategy to slow IBC progression.

In this study, we investigated the effectiveness of targeting the LIF/LIFR axis in IBC cells using EC359, a first-in-class LIFR inhibitor. Our results showed that LIFR inhibition using siRNA or pharmacological inhibition using EC359 inhibited the clonogenic potential of IBC cell lines. EC359 treatment reduced cell viability, reduced the invasive potential of IBC cells, and induced apoptosis. Mechanistic studies suggested that EC359 promotes IBC cell death by inducing ferroptosis. Using IBC cell-derived xenografts, we demonstrated EC359’s effectiveness in reducing tumor growth. Overall, our findings highlight the critical role of the LIF/LIFR signaling axis in IBC tumor growth and support the potential of EC359 as a therapeutic strategy for IBC.

## 2. Materials and Methods

### 2.1. Cell Culture and Reagents

KPL4 and SUM149 [13] cells were cultured in Dulbecco’s Modified Eagles Medium/Nutrient Mix F-12 Ham (1 µg/mL hydrocortisone, 5 µg/mL insulin, 2% Antibiotic/Antimycotic Solution (Corning, Corning, NY, USA), 1% Sodium Pyruvate (Corning), 5% FBS). SUM190 cells were purchased from BioIVT (Detroit, MI, USA) and cultured in Ham’s F-12 (Gibco, Waltham, MA, USA) media with 10% FBS (1 g/L bovine serum albumin, 5 mM ethanolamine, 1 µg/mL HEPES, 1 µg/mL hydrocortisone, 5 µg/mL insulin, 50 nM sodium selenite, 5 µg/mL apo-Transferrin, 10 nM triiodo-L-thyronine), 2% Antibiotic/Antimycotic Solution (Corning).

### 2.2. LIFR-Knockdown

IBC cells were transfected with LIFR-targeting siRNAs at final concentration of 20 nM (siLIFR-1: SASI_Hs02_00330114, ccagatactcctcaacaactg; and siLIFR-2: SASI_Hs02_00330115, aaatgttgcaatcaagattcg) or a non-targeting control siRNA (siRNA Universal Negative Control #1, Sigma-Aldrich, St. Louis, MO, USA) using Lipofectamine RNAiMAX (Invitrogen, Waltham, MA, USA) according to the manufacturer’s protocol. Briefly, siRNA and Lipofectamine RNAiMAX were separately diluted in Opti-MEM (Gibco), mixed, and incubated for 15 min at room temperature to allow complex formation. The transfection complexes were then added to cells at 50–70% confluency in antibiotic-free medium. After 6 h, the medium was replaced with complete growth medium, and cells were incubated for 60–72 h before further analysis. Efficient knockdown of LIFR expression was confirmed by Western blotting. A portion of the transfected cells was seeded into 6-well plates for colony formation assays and into Boyden invasion chambers for invasion assays.

### 2.3. Cell Viability, Clonogenicity, Apoptosis, and Invasion Assays

For the cell viability assays, IBC cells were plated at a density of 500 cells per well in a 96-well plate and treated with varying concentrations of EC359. Following a five-day incubation period, cell viability was quantified using the MTT assay. The clonogenicity was assessed by seeding cells at a low density (500 cells per well) and culturing them with EC359 or vehicle for 5 days. Subsequently, the medium was replaced with regular cell culture medium, and the cells were cultured for 2 weeks. The colonies were fixed with methanol, stained with 0.5% crystal violet, and the percentage area of the colonies was quantified using the Colony Area plugin in ImageJ software version 1.54p. Apoptosis was evaluated by treating cells with either the vehicle or EC359 (50 nM) for 24–48 h, followed by Annexin V and Propidium Iodide staining (FITC Annexin V Apoptosis Detection Kit with PI-BioLegend Catalog number-640914, San Diego, CA USA), as per established protocols [12]. Cell invasion was assessed using Boyden chamber assays with Matrigel-coated transwell inserts (8 µm pore size, Corning, NY, USA). Briefly, IBC cells were harvested, resuspended in serum-free medium and seeded into the upper chamber at a density of 1 × 10^5^ cells per well in 700 µL of serum-free medium containing EC359 (100 nM) or a vehicle control. The lower chamber was filled with 1000 µL of complete medium containing 20% FBS as a chemoattractant. After 22 h of incubation at 37 °C in a 5% CO₂ humidified incubator, non-invaded cells on the upper surface of the membrane were removed with a cotton swab. Invaded cells on the lower membrane surface were stained with 0.5% crystal violet and imaged using a light microscope. Cells in five random fields per membrane were counted, and invasion was quantified as the average number of invaded cells per field [12].

### 2.4. Western Blotting

Cells were lysed with RIPA buffer (Thermo Scientific, Waltham, MA, USA) plus EDTA and Halt Protease and Phosphatase inhibitors (Thermo Scientific). Lysates were centrifuged at 13,000 rpm for 20 min at 4 °C. Proteins were separated by SDS-PAGE (10 and 15%) and transferred onto nitrocellulose or PVDF membranes. Membranes were probed for LIFR (A-10) (1:500 sc-515337) or LIFR antibodies (C-19) (1:500 sc-659); β-actin (1:1000 Sigma A3854; CST 8457S); Vinculin (1: 1000 Sigma V9264); pSTAT3^Y705^ (1:1000 CST 9145S); STAT3 (1:1000 CST 4904S); pS6 S235/236 (1:1000 CST 4858S); S6 (1:1000 CST 22171L); pmTOR^S2448^ (1:1000 CST 5536S); mTOR (1:1000 CST 2972S); and GPX4 (1:1000 CST 52455S). The signal was detected and developed with ECL substrates (BioRad, Hercules, CA, USA) using secondary antibodies conjugated to horseradish peroxidase.

### 2.5. RT-qPCR

Cells were treated with either the vehicle or EC359 (100 nM) for 6 h. RNA was isolated using the TRIZOL method. cDNA was synthesized using the High-Capacity cDNA Reverse Transcription Kit. Real-time quantitative PCR utilized Powerup SYBRGreen Master Mix and standard conditions applied with 60 °C annealing temperature for all primers. All kits were obtained from Applied Biosystems (Waltham, MA, USA). Primer sequences are listed in Table 1.

### 2.6. Lipid Peroxidation

IBC cells were treated with vehicle control or EC359 (20 nM) for 12 h. After 10 h of EC359/vehicle incubation, Image-iT^®^ Lipid Peroxidation Sensor was spiked into the media for 2 h. Cells were processed using the manufacture’s protocol (Thermo Fischer, Waltham, MA, USA). Cell numbers were equalized across all samples and were analyzed using FACs Analysis as described.

### 2.7. In Vivo Xenograft Studies

All animal studies were performed in accordance with the approved IACUC procedure and guidelines of UT Health San Antonio. KPL4 tumor xenografts were established in the mammary fat pad of SCID female mice as described [12]. Following tumor establishment, mice were randomly assigned to receive either the vehicle or EC359 (5 mg/kg, intraperitoneally). The treatment protocol involved 5 consecutive days of drug administration, followed by a 2-day treatment break. Tumor growth was monitored biweekly using digital calipers. The tumor volume was determined using the formula: tumor volume = ½ (L × W^2^), where L represents the longitudinal diameter, and W denotes the transverse diameter. After treatment, mice were euthanized, and tumors were excised and prepared for subsequent histological analysis.

### 2.8. Immunohistochemistry

Sections were blocked using normal horse serum and incubated overnight for Ki-67 (Abcam 16667; 1:50, Cambridge, UK) and GPX4 (Proteintech 67763-1-Ig; 1:50, Rosemont, IL, USA) followed by incubation with ImmPRESS^®^ HRP Universal Antibody (Vector, Stuttgart, Germany) for 30 min. at room temp. DAB substrate (Vector) was used to visualize positive staining and counterstained with hematoxylin. Percent Ki-67 positive cells and GPX4 H-score were calculated from five randomly selected fields at 20×.

### 2.9. Statistical Analysis

The GraphPad Prism software 10 was employed to analyze the data. ANOVA or *t*-test were employed to ascertain statistical significance, with a *p*-value of less than 0.05 being considered significant.

## 3. Results

### 3.1. LIFR Knockdown Reduced the Clonogenicity and Invasion of IBC Cells

**Figure 1:**
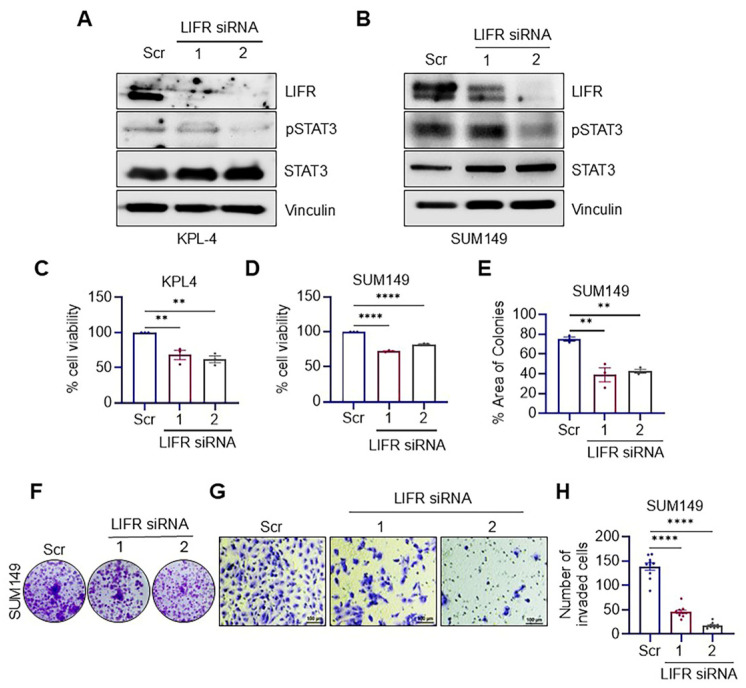
Genetic knockdown of LIFR suppresses the growth and invasion of IBC cells. Validation of LIFR knockdown in KPL4 (**A**) and SUM149 (**B**) cells was confirmed through Western blotting. The impact of LIFR knockdown using two distinct siRNAs on cell viability was assessed in KPL4 (**C**) and SUM149 (**D**) cells using the MTT assay. The effects of LIFR knockdown on SUM149 cell survival were evaluated through colony formation assays, with quantification of colony formation (**E**) and representative colony images (**F**) provided. The effect of LIFR knockdown on SUM149 cell invasion was analyzed using a Boyden chamber assay, with images of invaded cells (**G**) and their quantification (**H**) were shown. Original uncropped Western blots are provided in the Supplementary Materials. Data are represented as mean ± SE. ** *p* < 0.01; **** *p* < 0.0001.

To evaluate the significance of the LIFR signaling pathway in IBC cells, we initially employed a genetic knockdown approach utilizing two siRNAs that uniquely target separate regions of LIFR mRNA. Western blot analysis verified the effective knockdown of LIFR by both siRNAs (Figure 1A,B and Figure S1A,B). The knockdown of LIFR significantly reduced the cell viability (Figure 1C,D) and the clonogenicity of IBC cells in the colony formation assays (Figure 1E,F), demonstrating reduced cell growth under LIFR knockdown conditions. Given the invasive characteristics of IBC and the established involvement of LIFR in downstream signaling related to invasion, we assessed the effect of LIFR knockdown on the invasive potential of IBC cells. In the Boyden chamber assay, LIFR knockdown greatly diminished the invasion potential of IBC cells (Figure 1G,H). Collectively, these results indicate that LIFR plays a significant role in the growth and invasion of IBC cells.

### 3.2. LIFR Inhibitor EC359 Reduced the Cell Viability, Clonogenicity and Invasion of IBC Cells

**Figure 2:**
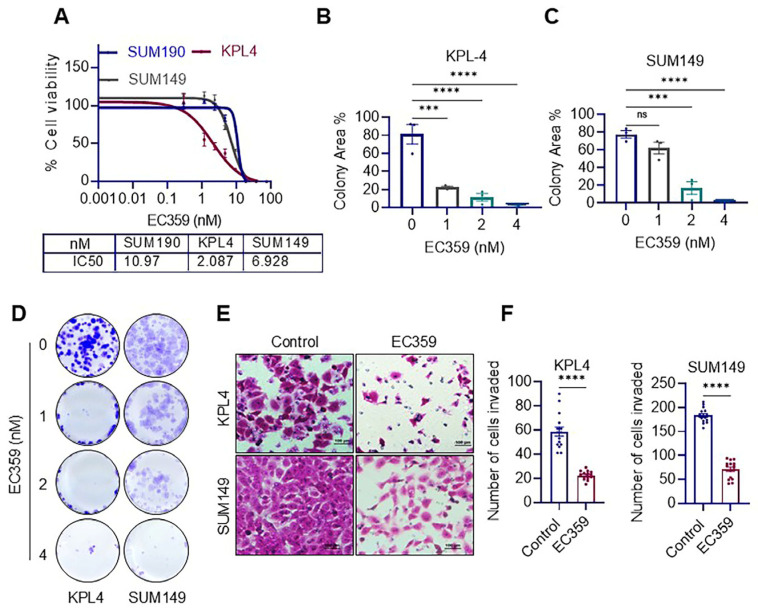
EC359, a LIFR inhibitor, decreased the viability, clonogenicity, and invasion of IBC cells. (**A**) The impact of EC359 on the viability of IBC cells (SUM190, KPL4, SUM149) was determined using MTT assay. (**B**–**D**) EC359’s impact on the clonogenic potential of IBC cells was assessed using the colony formation assay. The quantification of the percentage of colonies (**B**,**C**) and images (**D**) are shown. (**E**,**F**) EC359’s impact on the invasion of IBC cells was assessed through Boyden chamber assays. In panel (**E**), images are displayed, and panel (**F**) displays a quantification of the percentage of cells that were invaded. The scale bar represents 100 µm. Data are represented as mean ± SE. ns, not significant; *** *p* < 0.001; **** *p* < 0.0001.

We then evaluated the importance of LIFR signaling by employing the recently developed LIFR pharmacological inhibitor EC359. The LIFR inhibitor EC359 is a potent, high-affinity, and selective agent with a Kd of 10.2 nM. Its specificity to LIFR was illustrated in our previous study using siRNA [12]. It effectively prevents LIF/LIFR interactions by directly interacting with LIFR [12]. In the MTT-based cell viability assay, EC359 exhibited a dose-dependent reduction in all three IBC cell lines (KPL4, SUM149, SUM190) that were examined with IC_50_ values between ~2–10 nM (Figure 2A). Additionally, the clonogenicity of the KPL4 and SUM149 cell lines was substantially reduced by EC359, which further corroborated the results of the cell viability experiments (Figure 2B–D). In the Boyden chamber assays, EC359 significantly inhibited the invasive capacity of IBC cells compared to control vehicle-treated cells (Figure 2E,F). These findings collectively demonstrate the susceptibility of IBC cells to the LIFR inhibitor EC359.

### 3.3. EC359 Reduced the Activation of LIFR Down Stream Signaling

**Figure 3:**
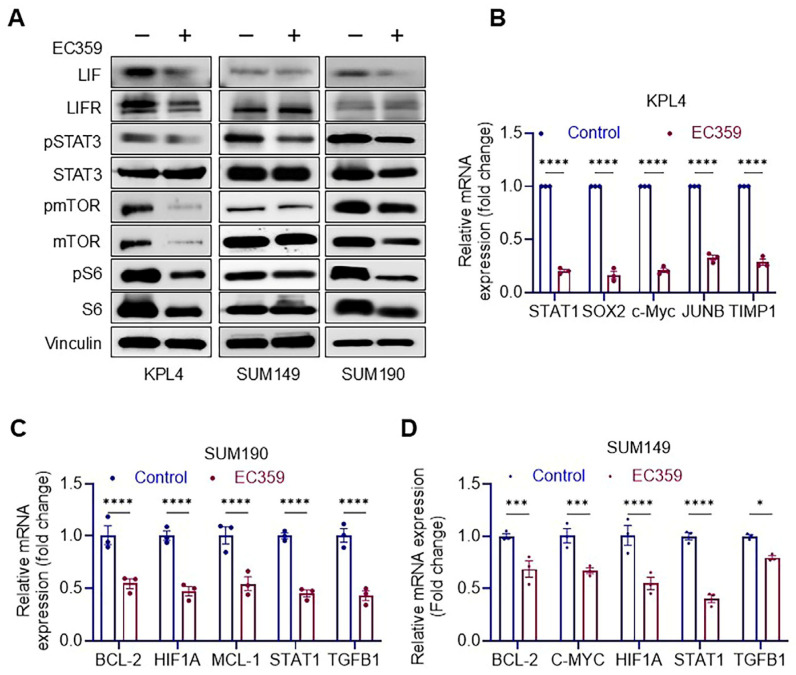
(**A**), Immunoblot analysis showing the suppression of downstream targets in response to EC359 (100 nM) treatment for 6 h. (**B**–**D**), RT-qPCR analysis demonstrating the downregulation of transcriptional targets regulated by LIFR following EC359 (100 nM) treatment for 6 h in three IBC model cells. Original uncropped Western blots are provided in the Supplementary Materials. Data are represented as mean ± SE. * *p* < 0.05; *** *p* < 0.001; **** *p* < 0.0001.

We subsequently established the mechanisms of action through which EC359 diminishes IBC cell viability. The Western blotting analyses demonstrated that EC359 significantly suppressed STAT3 and subsequent downstream signaling components, as indicated by a decrease in the phosphorylation levels of the corresponding targets (mTOR, and S6) (Figure 3A and Figure S2A–C). Given that LIFR signaling activates several genes, we investigated the changes in the expression levels of several LIFR target genes using RT-qPCR (Figure 3B–D). The findings indicated a substantial decrease in the LIFR target genes following EC359 therapy. The data suggest that EC359 inhibits IBC cells by targeting STAT3 signaling and downregulating LIFR target genes.

### 3.4. EC359 Induced Apoptosis in IBC Cells Through the Ferroptosis Mechanism

**Figure 4:**
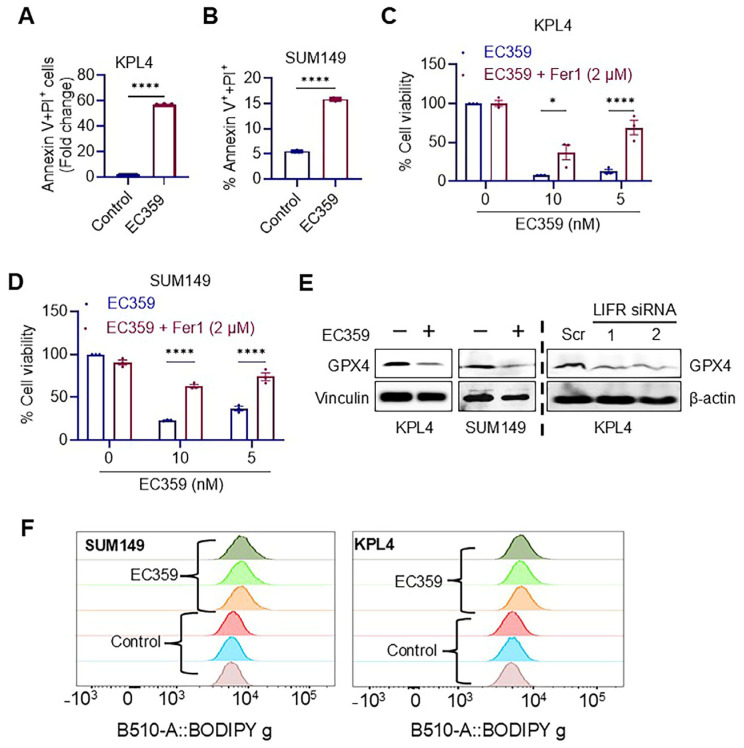
EC359 triggered apoptosis in IBC cells via the ferroptosis pathway. Annexin V^+^ and PI^+^ IBC cells KPL4 (**A**) and SUM149 (**B**) treated for 48 h for KPL4 and 25 h. in the case of the SUM149 cell line, with EC359 (50 nM). Cell viability assay of IBC cells KPL4 (**C**) and SUM149 (**D**) treated with varied doses of EC359 alone or combination with 2 µM ferrostatin-1. (**E**) Western blot analyses of ferroptosis marker GPX4 in response to control or EC359 (100 nM) for 6 h treatment and by knocking down LIFR through siRNA. (**F**) Lipid peroxidation measured by flow cytometry using Image-iT^®^ in IBC cells treated for 12 h at 20 nM EC359 or control. Original uncropped Western blots are provided in the Supplementary Materials. Data are represented as mean ± SE. * *p* < 0.05; **** *p* < 0.0001.

We subsequently investigated the apoptotic effects of EC359 in IBC cells through an Annexin V assay. The results demonstrated that, in comparison to controls, EC359 treatment markedly elevated the number of Annexin V-positive cells (Figure 4A,B and Figure S3A,B) in both the KPL4 and SUM149 cell lines. Recent studies indicate that the suppression of LIF/LIFR autocrine loops in ovarian cancer induces cell death by triggering ferroptosis through the downregulation of GPX4 levels [14]. To investigate whether EC359 enhances ferroptosis, we utilized ferrostatin-1 (Fer-1), a well-characterized inhibitor of ferroptosis which serves as a lipophilic radical scavenger. The addition of Fer-1 effectively mitigated the cytotoxic effects of EC359 on IBC cells (Figure 4C,D). To clarify the mechanism via which EC359 triggers ferroptosis in IBC cells, we investigated GPX4, a physiological inhibitor of ferroptosis and a contributor to lipid peroxidation detoxification. Western blot analysis demonstrated a decrease in GPX4 protein levels in IBC cells treated with EC359 and in LIFR KD cells (Figure 4E and Figure S3C). Lipid peroxidation is a hallmark of ferroptosis. Flow cytometric analysis of lipid peroxidation in IBC cells, with and without EC359 treatment, confirmed the oxidation of the BODIPY™ 581/591 C11 reagent. Collectively, these results suggest that EC359-mediated cell death occurs via ferroptosis (Figure 4F).

### 3.5. LIFR Inhibitor EC359 Reduced the Growth of IBC Xenografts

**Figure 5:**
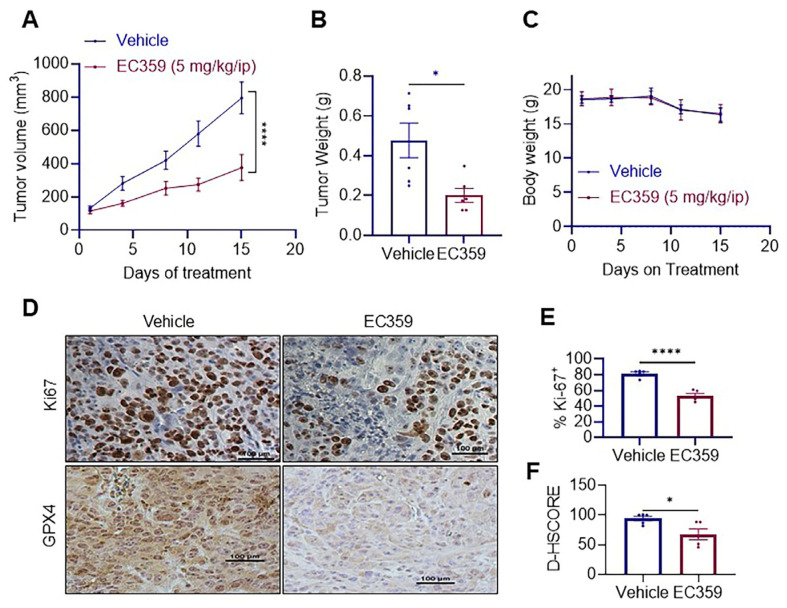
(**A**) In vivo tumor growth of KPL4 IBC cell line in response to the treatment with vehicle or EC359 (5 mg/kg/i.p.). (**B**) Tumor weights of harvested tumors treated with vehicle or EC359 treatment. (**C**) Body weight of mice during drug treatment. (**D**) IHC images of ki-67 and GPX4 shown in the left panel and (**E**,**F**) quantification of Ki-67 and GPX levels shown as bar graphs in the right panel. Data are represented as mean ± SE. * *p* < 0.05; **** *p* < 0.0001.

To evaluate the efficacy of LIFR inhibition in vivo, we established KPL4 cell-derived xenografts in SCID mice. After the establishment of xenografts, the mice were randomized into two groups: (1) vehicle and (2) EC359 therapy (5 mg/kg, intraperitoneally, five days per week). The results indicated that EC359 administration markedly reduced tumor volume and size compared to control mice, with no impact on body weight (Figure 5A–C). Additionally, IHC analysis of vehicle and EC359 treated tissues using Ki-67 and GPX4 antibodies revealed decreased proliferation and reduced GPX4 levels in EC359-treated tumor tissues (Figure 5D–F). Collectively, these results indicate that the LIFR inhibitor EC359 has the ability to reduce the growth of IBC in vivo.

## 4. Discussion

IBC is a rare subtype of breast cancer that accounts for approximately 7% of breast cancer-related deaths [5]. To date, no specific targeted therapy treatments have been approved for IBC patients. This study is focused on the identification of a novel targeted therapy for IBC to address this knowledge gap. Our study indicates that the LIF/LIFR axis is an essential pathway in IBC. We have shown that the growth and invasion of IBC cells are reduced by blocking LIFR signaling using genetic knockdown and pharmacological inhibitions. LIFR inhibition was demonstrated to induce cell death through ferroptosis. The LIF/LIFR axis significantly regulates the activation of STAT3 and its downstream signaling pathways in IBC cells. Further, the in vivo progression of IBC is effectively reduced by the inhibition of LIFR with EC359. These results suggest that LIFR may be a potential therapeutic target for IBC.

LIF/LIFR signaling has been associated with proliferation, progression, metastases, stemness, and chemoresistance in several cancers [10,11,15,16,17,18,19]. LIFR signaling is regulated through a complex formed by LIFR and glycoprotein 130 (gp130) [15]. Although LIFR lacks intrinsic tyrosine kinase activity, LIFR and gp130 form a stable complex with the JAK-Tyk family of cytoplasmic tyrosine kinases. When LIF binds to this complex, it activates a cascade of signaling pathways that include STAT, AKT, and mTOR, all of which are involved in the progression of breast cancer [11,15,16]. The JAK-STAT3 pathway is becoming increasingly significant in the progression of IBC [8]. Molecular profiling studies identified JAK-STAT3 signaling as an important regulator of IBC cell growth [9]. In this study, using genetic knockdown and pharmacological inhibition, we showed that LIFR played a critical role in the activation of STAT3 signaling in IBC cells. Further, RT-qPCR analyses also confirmed that LIFR inhibition reduced the expression of STAT3 target genes.

Autocrine or paracrine loops of LIF/LIFR have been identified in several cancers, such as pancreatic, lung, and ovarian cancer [14,20,21,22]. Overexpression of LIF has been shown to initiate autocrine signaling, thereby driving the progression of breast cancer cells [23]. The potential of inhibiting the LIF/LIFR axis for cancer treatment has been demonstrated using neutralizing antibodies [20] or engineered ligand traps [24]. A novel small molecule inhibitor, EC359, has recently been identified, which selectively binds to LIFR and inhibits LIF binding. EC359 is effective only on cells that express LIF and LIFR [12]. For example, EC359 had little efficacy against ER+ breast cancer cells, such as MCF7, which express low amounts of LIF and LIFR. Conversely, TNBC models like MDA-MB-231 cells exhibit elevated levels of LIF and LIFR and demonstrate significant responsiveness to EC359. The ablation of LIFR in the TNBC model MDA-MB-231 cells significantly diminishes the efficacy of EC359 [12]. Other studies have also shown the effectiveness of EC359 in inhibiting pancreatic cancer [25,26], renal cancer [27], and ovarian cancer [14]. This study, employing three distinct IBC cell lines and cell-derived xenografts, demonstrates that EC359 significantly diminishes IBC cell survival and tumor growth in vivo.

Ferroptosis is a unique type of cell death defined by iron-dependent phospholipid peroxidation [28]. Pharmacological targeting of ferroptosis presents significant potential as an anticancer approach [29]. One of the primary defense mechanisms against ferroptosis involves the glutathione peroxidase 4 (GPX4)–reduced glutathione (GSH) system [30]. Recent studies have demonstrated that LIFR inhibition with EC359 induces ferroptosis in renal cancer cells by inactivating GPX4 and depleting GSH [27]. Similarly, EC359 has been shown to induce ferroptosis by downregulating GPX4 levels in ovarian cancer cells. In this study, we observed that the ferroptosis inhibitor Fer-1 significantly reduced EC359-induced cell death. Furthermore, our results show that EC359 downregulates GPX4, disrupting the GSH-mediated defense system, which triggers lipid peroxidation and ultimately leads to the death of IBC cells. These findings are consistent with two recent studies suggesting that LIFR downstream signaling pathways contribute to the suppression of the ferroptosis defense system, and that inhibiting LIFR signaling may promote cell death via ferroptosis.

## 5. Conclusions

In conclusion, our findings indicate that the LIF/LIFR signaling axis is functional in IBC cells. The growth and invasion of IBC cells are reduced by the reduction in LIFR expression through siRNA or pharmacological inhibition. EC359 effectively inhibited LIFR signaling and decreased the viability of IBC cells in vitro and in vivo. LIFR inhibition activates apoptosis in IBC cells by promoting ferroptosis. Since EC359 is a small molecule inhibitor with favorable pharmacokinetics and bioavailability, it can be utilized to treat IBC either as a monotherapy or in conjunction with existing standard treatment protocols.

## Figures and Tables

**Table 1:** List of primer sequences.

Gene Name	Forward Primer (5′→3′)	Reverse Primer (5′→3′)
STAT1	CAGCTTGACTCAAAATTCCTGGA	TGAAGATTACGCTTGCTTTTCCT
SOX2	TGCGAGCGCTGCACAT	TCATGAGCGTCTTGGTTTTCC
c-Myc	GGCTCCTGGCAAAAGGTCA	CTGCGTAGTTGTGCTGATGT
JUNB	ACGACTCATACACAGCTACGG	GCTCGGTTTCAGGAGTTTGTAGT
TIMP1	CTTCTGCAATTCCGACCTCGT	ACGCTGGTATAAGGTGGTCTG
BCL-2	GGTGGGGTCATGTGTGTGG	CGGTTCAGGTACTCAGTCATCC
HIF1A	CACCACAGGACAGTACAGGAT	CGTGCTGAATAATACCACTCACA
MCL-1	GTAATAACACCAGTACGGACGG	CCACAAACCCATCCTTGGAAG
TGFB1	CAATTCCTGGCGATACCTCAG	GCACAACTCCGGTGACATCAA
18S rRNA	GCTTAATTTGACTCAACACGGGA	AGCTATCAATCTGTCAATCCTGTC

## Data Availability

This article contains all of the data that were used in this investigation.

## Supplementary Materials

The following supporting information can be downloaded at: https://www.mdpi.com/article/10.3390/cancers17050790/s1, Figures S1–S3: Uncropped original blots for Western blot data used in the main text.
