# Supplementary figures and images for: Targeting the Leukemia Inhibitory Factor/Leukemia Inhibitory Factor Receptor Axis Reduces the Growth of Inflammatory Breast Cancer by Promoting Ferroptosis
Source: Cancers (Basel). 2025 Feb 25;17(5):790. doi: 10.3390/cancers17050790 (PMC11898489; doi:10.3390/cancers17050790)

# Figure S1A

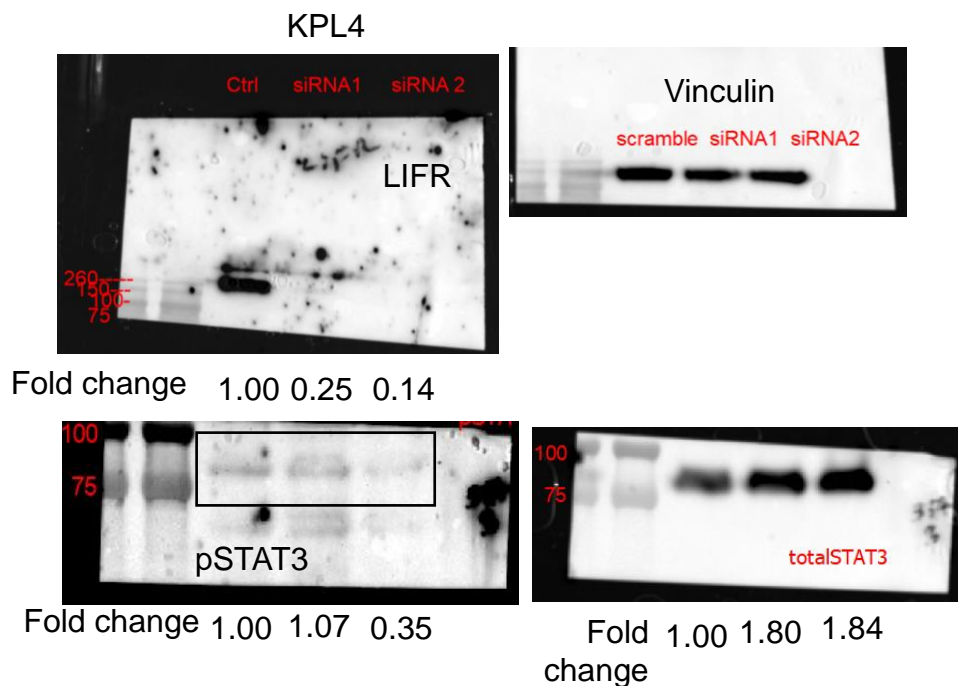

# Figure S1B

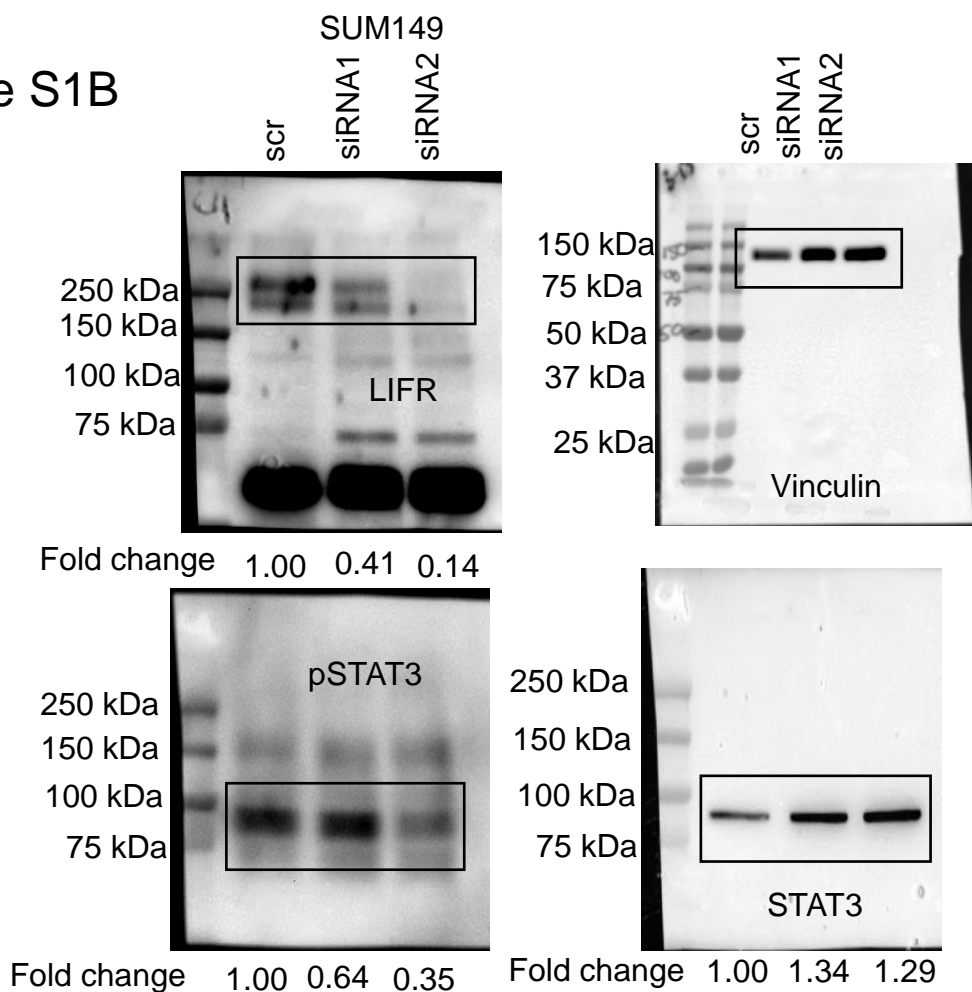

Figure S2A

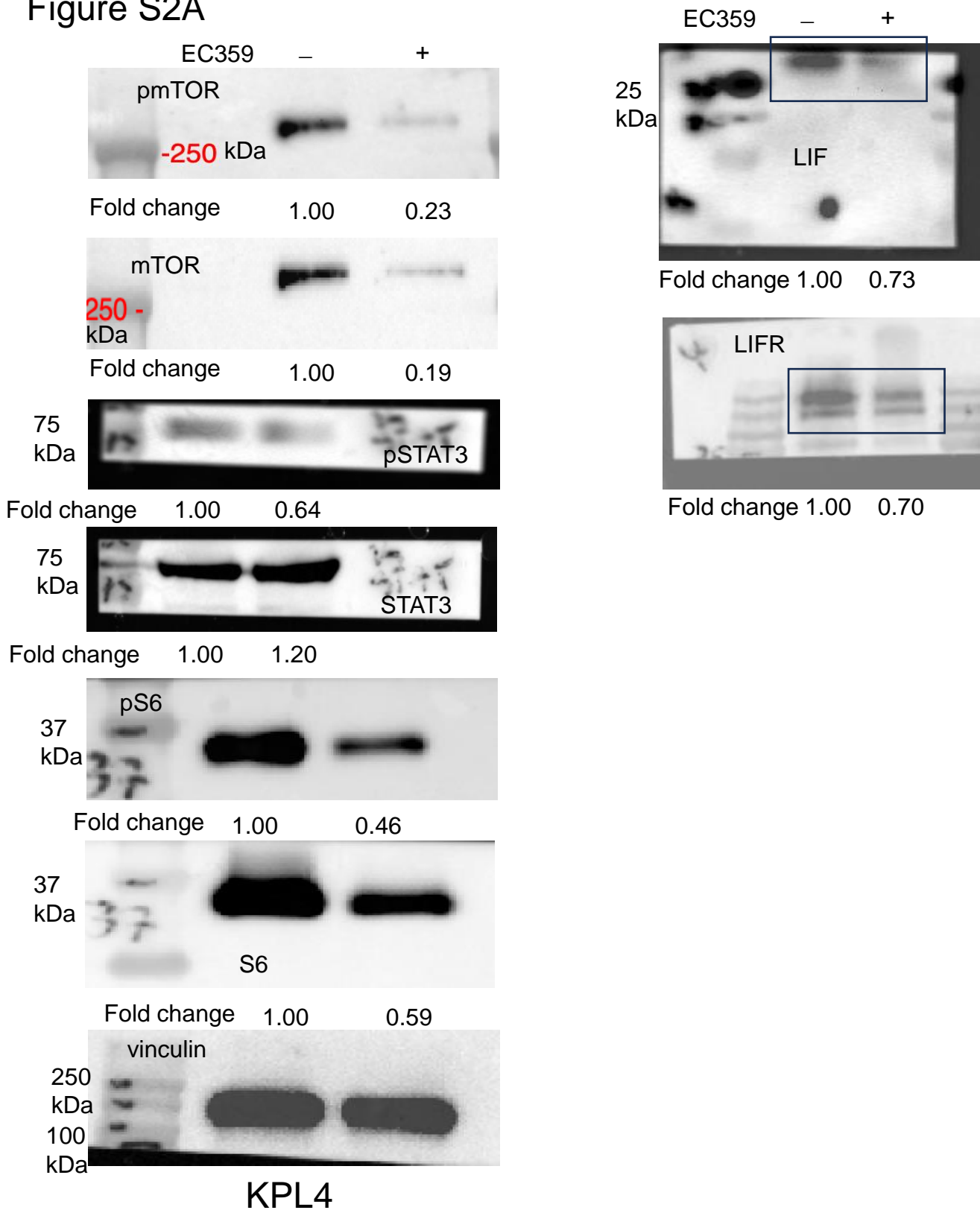

Figure S2B

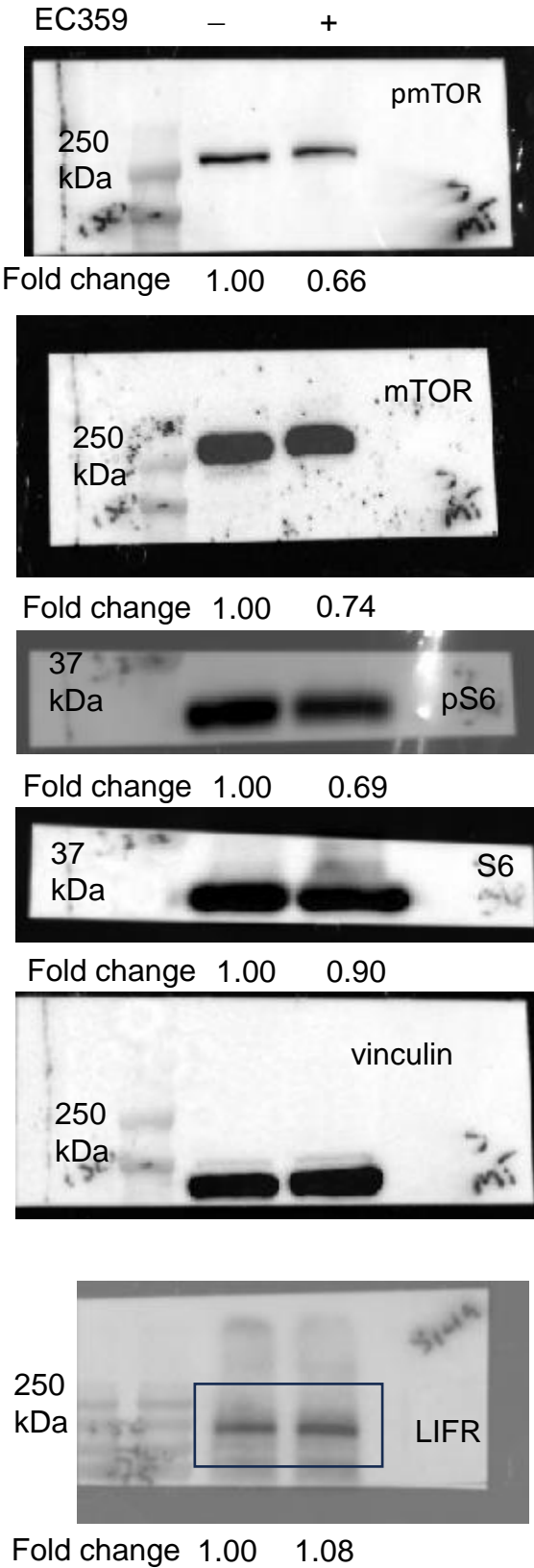

SUM149

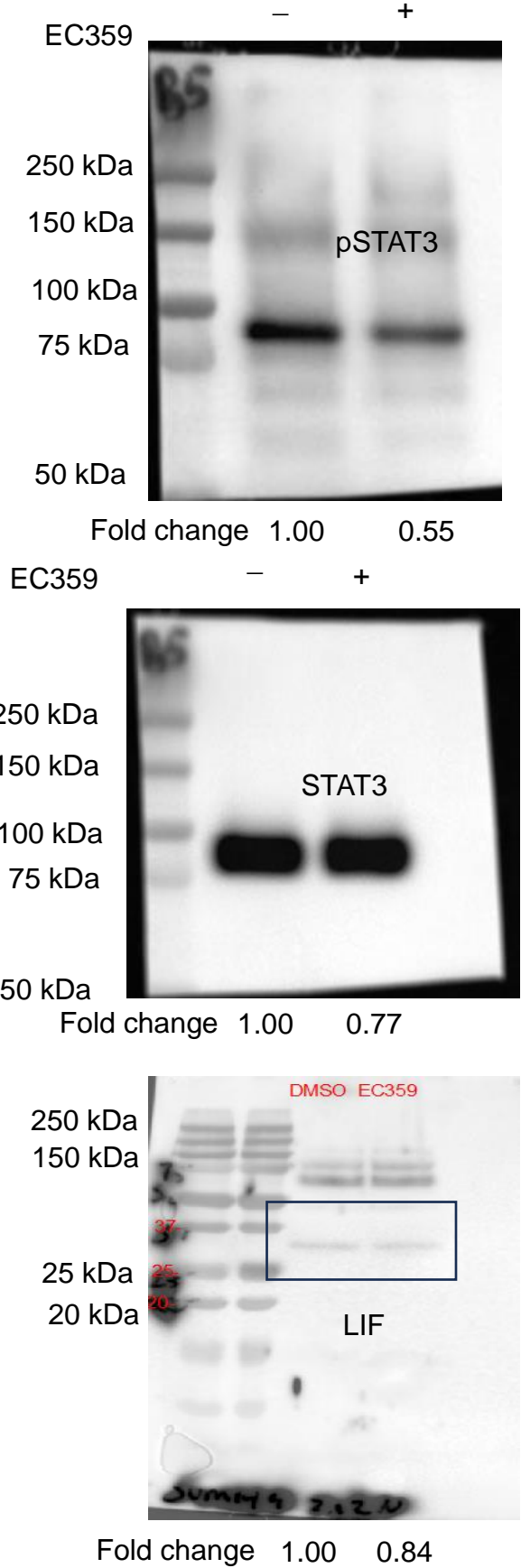

Figure S2C

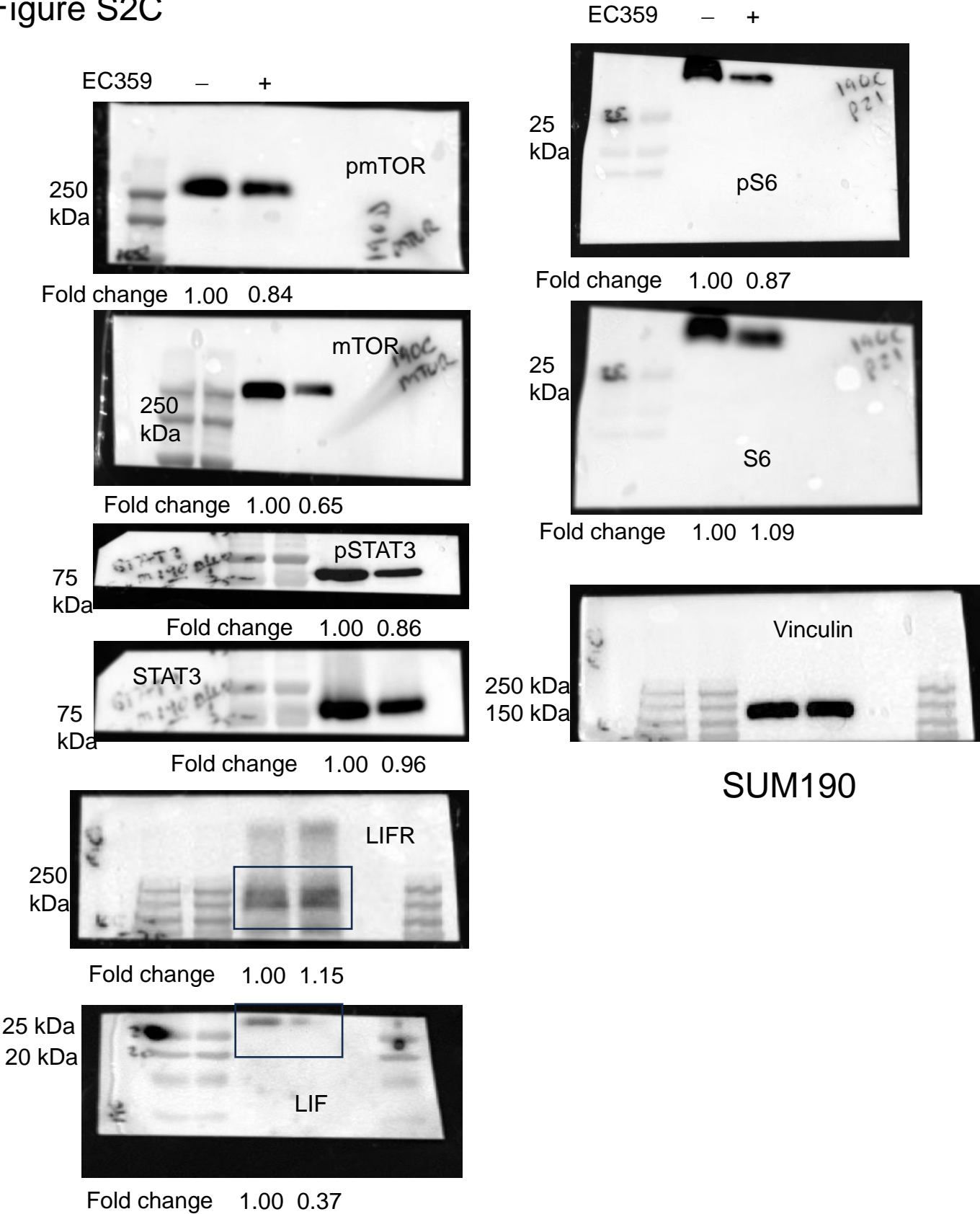

Figure S3A

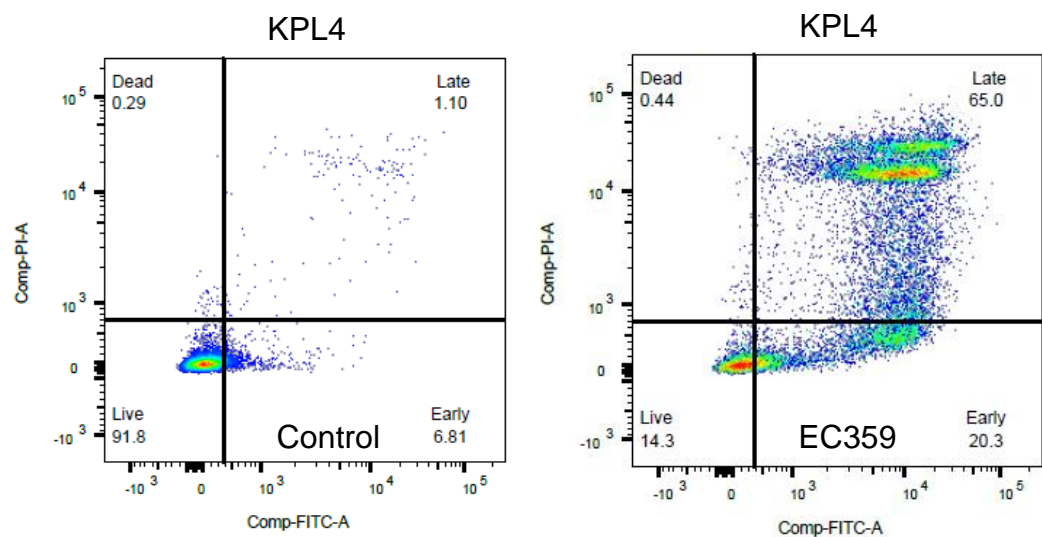

Fig. S3B

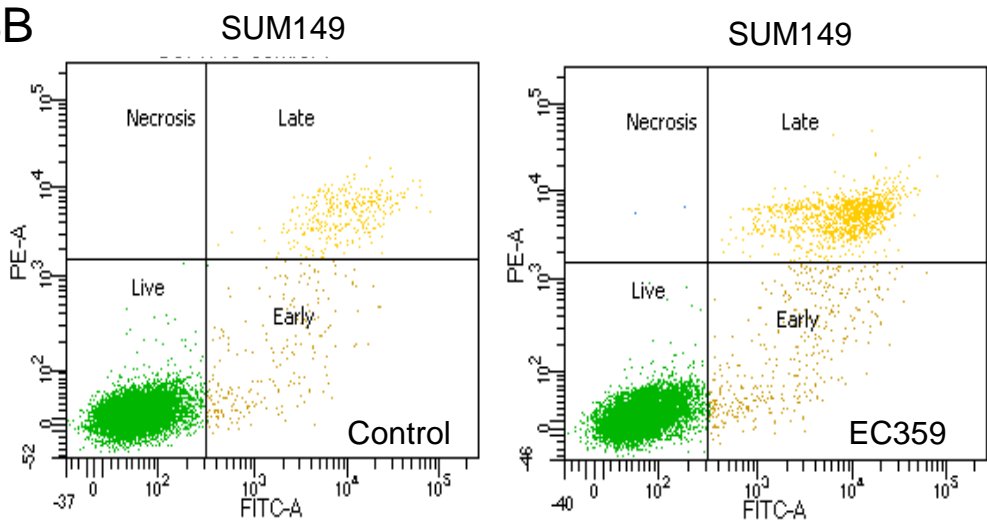

Figure S3C

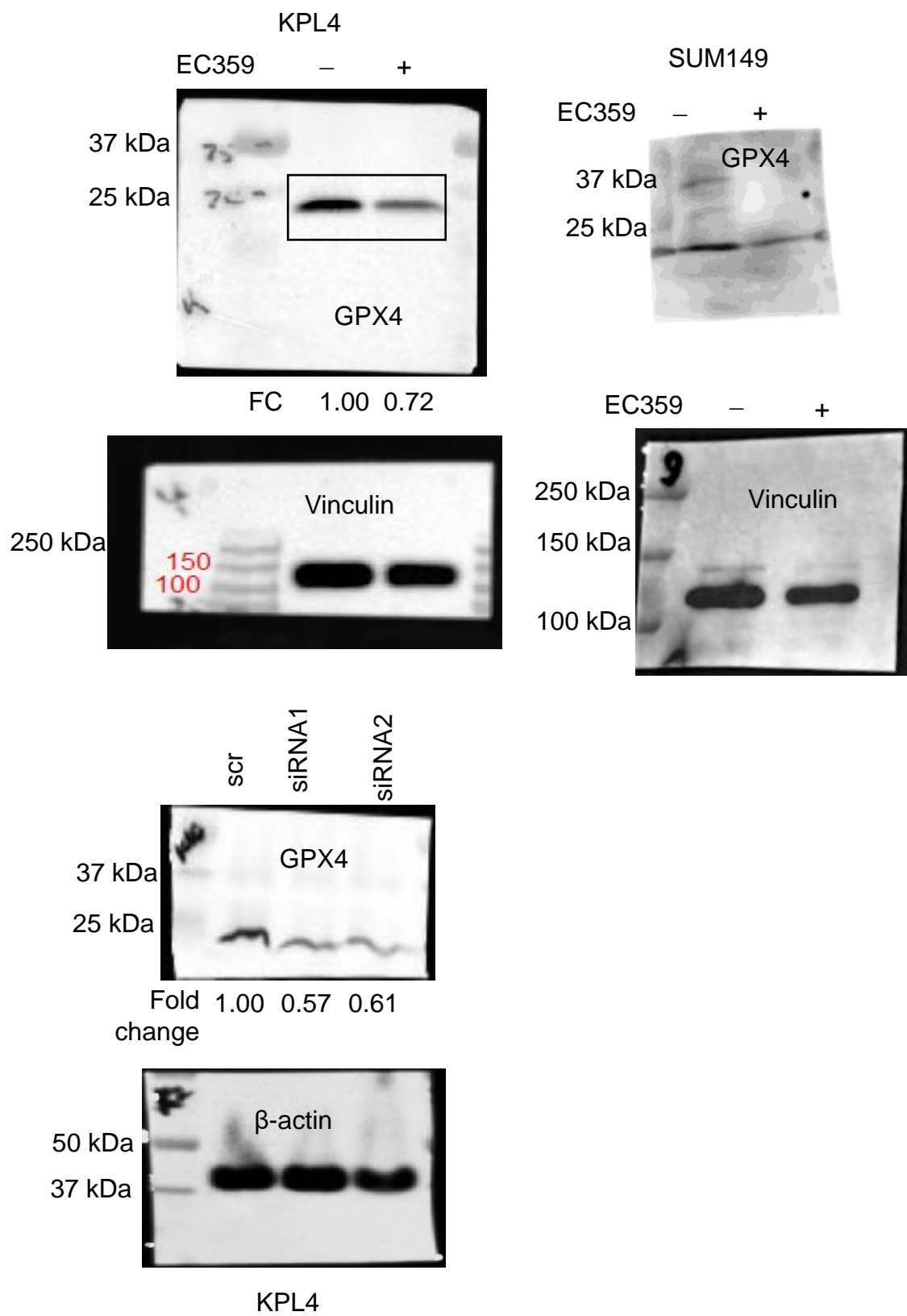

Supplement: Supplementary file 1 [file cancers-17-00790-s001.zip › cancers-3432090-supplementary.pdf]
